# Identification and field bioassays of the sex pheromone of *Eurytoma maslovskii* (Hymenoptera: Eurytomidae)

**DOI:** 10.1038/s41598-020-67252-7

**Published:** 2020-06-24

**Authors:** Chang Yeol Yang, Kenji Mori, Junheon Kim, Ki Bong Kwon

**Affiliations:** 1 Horticultural and Herbal Crop Environment Division, National Institute of Horticultural and Herbal Science, Wanju, Republic of Korea; 2 Photosensitive Materials Research Center, Toyo Gosei Co., Ltd, 4-2-1 Wakahagi, Inzai-shi, Chiba 270-1609 Japan; 3 Forest Insect Pests and Diseases Division, National Institute of Forest Science, Seoul, Republic of Korea; 4 AD Corporation, Andong, Gyeongsangbuk-do Republic of Korea

**Keywords:** Ecology, Chemistry

## Abstract

Long-range sex pheromones are generally considered to be a main cue for mate recognition in the order Hymenoptera. Although considerable attention has been given to the identification of semiochemicals in the superfamily Chalcidoidea, which comprises 19 families, no study has identified active components in a field bioassay. We herein report the sensitive and selective pheromone communication system of the Korean apricot wasp, *Eurytoma maslovskii* (Eurytomidae), whose larvae feed on *Prunus mume* seeds. Using gas chromatography coupled with electroantennographic detection (GC-EAD) and mass spectrometry (GC-MS), we detected 2,10-dimethyldodecyl propionate and 2,8-dimethyldecyl propionate in thoracic extracts of female *E. maslovskii* at a ratio of 8:2 as the active pheromone components. Field experiments showed that the attractive effect of the two compounds is highly enantioselective. Racemic 2,10-dimethyldodecyl propionate and 2,8-dimethyldecyl propionate were not attractive to *E. maslovskii* males. In bioassays with single enantiomers, the (2 *S*,10 *R*)-enantiomer was highly attractive to male wasps, and the (2 *S*,8 *S*)-enantiomer was also attractive, although to a lesser degree. No synergistic effect between (2 *S*,10 *R*)- and (2 *S*,8* S*)-enantiomers was identified, and the (2 *S*,10 *R*)-enantiomer alone caught significantly more males than the natural pheromone extracts. The addition of other enantiomers to the (2 *S*,10* R*)-isomer significantly decreased the attraction of conspecific males. In addition, a very low dose of synthetic pheromone attracted conspecific males, showing that both female signaling and male response traits may have evolved to contribute to species-specific sexual communication in this species.

## Introduction

Long-range sex pheromones seem to be the most significant cues in sex attraction and recognition in Hymenoptera, as in Lepidoptera and Coleoptera^1^. Although considerable attention has been given to the identification of semiochemicals in hymenopteran species, the female long-range sex pheromones of only five species from the suborder Symphyta and six species from the suborder Apocrita have been completely characterized in field bioassays^2^.

The identified compounds include (*E*)-9-oxo-2-decenoic acid in *Apis mellifera* (Apidae)^3^, 3,7-dimethylpentadecan-2-yl acetate in *Neodiprion lecontei* (Diprionidae)^4^, (*Z*)-10-nonadecenal in *Pikonema alaskensis* (Tenthredinidae)^5^, ethyl (*Z*)-9-hexadecenoate in *Syndipnus rubiginosus* (Ichneumonidae)^6^, (3 *S*,5 *R*,6 *S*)-3,5-dimethyl-6-(methylethyl)-3,4,5,6-tetrahydropyran-2-one in *Macrocentrus grandii* (Braconidae)^7^, (*Z*,*Z*)-9,12-octadecadienal in *Ascogaster quadridentata* (Braconidae)^8^, (*R*,*Z*)-9-octadecen-4-olide in *Janus integer* (Cephidae)^9^, esters of 3,7-dimethyltridecan-2-ol in *Diprion pini* (Diprionidae)^10^, (*Z*)-6,14-pentadecadienal in *Acantholyda erythrocephala* (Pamphiliidae)^11^, 2-butyl-3,5-dimethyl pyrazine and 2-hydroxymethyl-3,5-diethyl-6-methylpyrazine in *Zaspilothynnus trilobatus* (Thynnidae)^12^, and 4-oxo-octanoic acid and 4-oxo-decanoic acid in *Vespa velutina* (Vespidae)^13^.

The superfamily Chalcidoidea comprises 19 families and contains approximately 22,500 known species, with an estimated total diversity of more than 500,000 species^14^. Evidence related to long-range sex pheromones has been found for chalcidoid species from Aphelinidae^15^, Chalcididae^16^, Pteromalidae^17^, Eulophidae^18^, Eurytomidae^19,20^, and Trichogrammatidae^21^. However, none of these studies identified the active components using field bioassays.

The structural elucidation of the sex pheromone of *Eurytoma amygdali* Enderlein (Eurytomidae), a serious pest of almonds in Europe and the Middle East, has been a widely explored issue since this pheromone was first described approximately five decades ago^19^. Krokos *et al.*^22^ identified a two-component sex pheromone consisting of (*Z,Z*)-6,9-tricosadiene and (*Z,Z*)-6,9-pentacosadiene in whole body extracts of female *E. amygdali* that attracted conspecific males. However, Duval and Millan^23^, in field bioassays, found that the two alkadienes were completely inactive.

Here, we investigated the female sex pheromone communication system of *Eurytoma maslovskii* Nikolskaja, whose larvae feed on Korean apricot, *Prunus mume* Sieb. et Zucc^24^ using GC-EAD, GC-MS analysis and field bioassays. We demonstrate that female *E. maslovskii* produce 2,10-dimethyldodecyl propionate and 2,8-dimethyldecyl propionate at very low levels and that conspecific males selectively respond to (2*S*,10*R*)- and (2*S*,8*S*)-enantiomers in the field.

## Results

**Figure 1:**
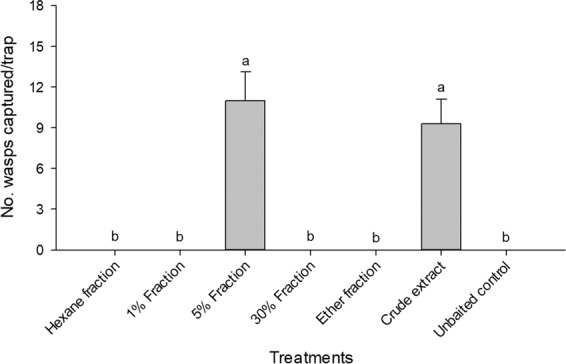
Number (mean + SE) of male *Eurytoma maslovskii* captured in traps baited with various fractions of thoracic extract obtained from conspecific females in Korean apricot orchards in Suncheon, Korea, on 4–8 May 2018 (*N* = 3 per treatment). There was statistical difference among treatments (Kruskal-Wallis analysis, *x*^2^ = 19.41, P = 0.0035). Bars with the same letter are not significantly different based on pairwise comparisons using a Mann-Whitney U-test (*P* > 0.05).

**Figure 2:**
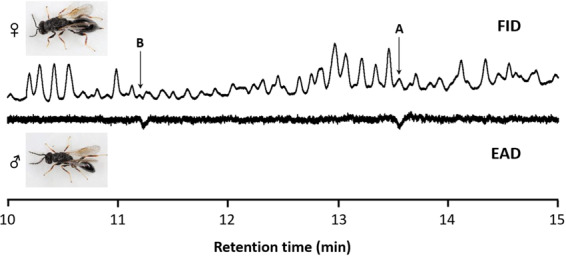
Male *Eurytoma maslovskii* antennal response (EAD) to compounds in the 5% fraction from female thoracic extracts, eluting from DB-5 cloumn (FID). Images of wasp adults were taken by one of the authors (CYY).

**Figure 3:**
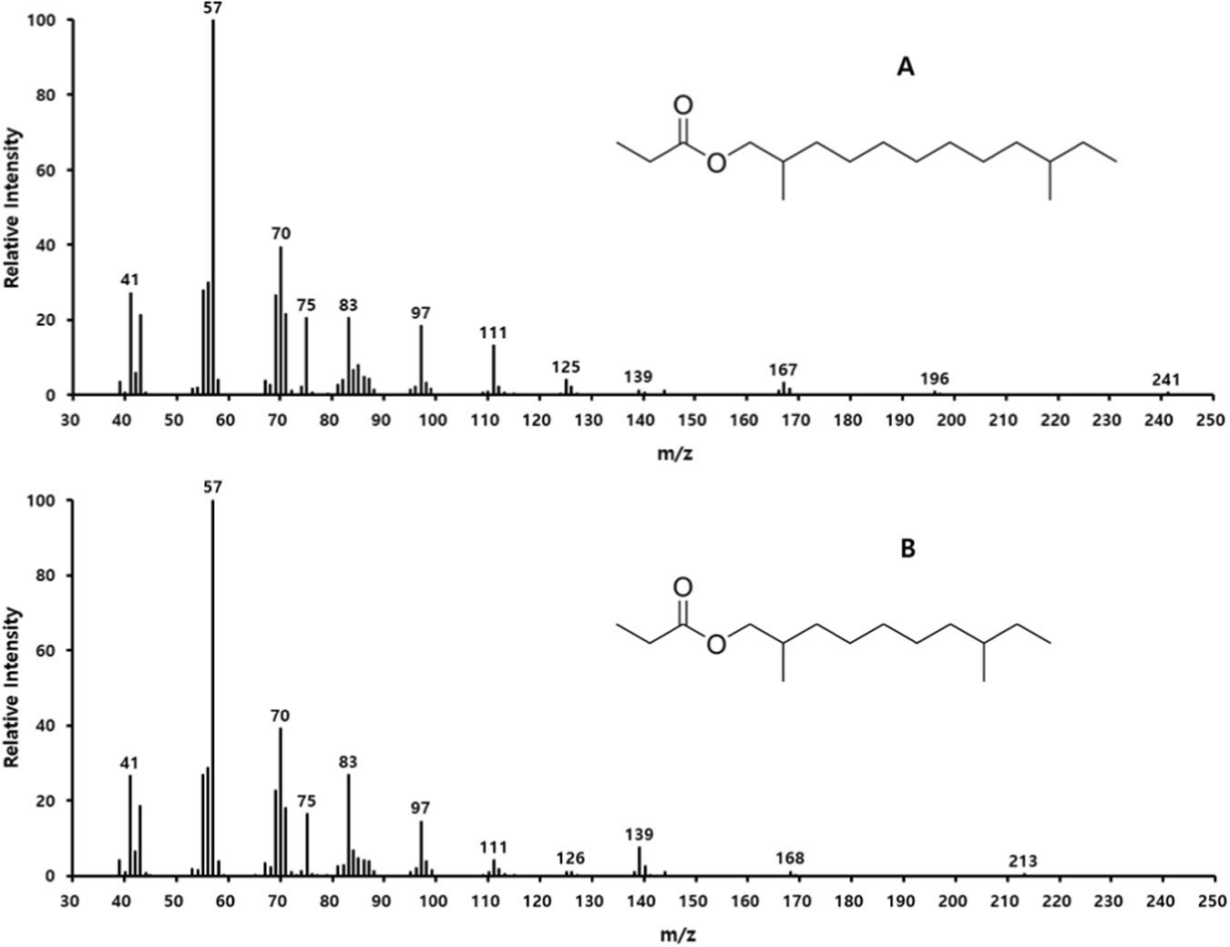
Electron impact ionization (70 eV) mass spectra of (**A**) the major pheromone component, 2,10-dimethyldodecyl propionate and (**B**) the minor component, 2,8-dimethyldecyl propionate.

To isolate the sex pheromone of *E. maslovskii*, the thoracic extracts of virgin females were fractionated on silica gel. When the extracts were fractionated into hexane, 1%, 5%, 30%, and diethyl ether fractions, only the 5% fraction was attractive to males in field experiment 1 (Fig. 1). There were no differences in the responses of male wasps to 5% fraction compared to the crude extract, indicating that this fraction was responsible for the attractiveness of the crude extracts. The GC-EAD analysis showed that male wasp antennae responded consistently to only two compounds in the 5% fraction of thoracic extracts obtained from virgin females (Fig. 2, peaks A and B). The mass spectrum of compound A (Fig. 3A) suggested that it was a propionate, as evidenced by the diagnostic peak at *m/z* 75 ([CH_3_CH_2_COOH_2_]^+^), indicative of a propionate functionality. The largest ion (M^+^-74) at *m/z* 196 indicated that the compound was a propionate with a molecular weight of 270. The comparatively strong peak at *m/z* 167 suggested the possibility of monomethyl branching at either the 3 or 11 position of alcohol moiety with 13 carbon chain, or of dimethyl branching at either the 3,X or X,10 position of alcohol moiety with 12 carbon chain. To identify compound A, the retention indices (RIs) of the compound in extract were compared with those of two C13 monomethyl branched propionates and five C12 dimethyl branched propionates using the same analytical methods. The RI values for compound A coincided with 2,10-dimethyldodecyl propionate (2,10-DiMe-12Pr) standard (RIs of 1812 on the DB-5MS column, and 2035 on the DB-Waxetr column), but different from those for the other six compounds (Table 1). In addition, the mass spectrum of the natural compound was similar to that of synthetic 2,10-DiMe-12Pr.

**Table 1:** Retention indices of the electroantennographic detector (EAD)-active compound (Peak A and B) from the 5% fraction of the thoracic extracts of female *Eurytoma maslovskii* and synthetic pheromone candidates on DB-5MS and DB-Waxetr columns.

Compound	DB-5MS	DB-Waxetr
**Female thoracic extract**		
Peak A	1812	2035
Peak B	1610	1831
**Synthetic pheromone candidates**		
2,8-dimethyldecyl propionate	1610	1831
3,7-dimethyldodecyl propionate	1792	2003
3,9-dimethyldodecyl propionate	1801	2025
2,10-dimethyldodecyl propionate	1812	2035
4,10-dimethyldodecyl propionate	1818	2049
6,10-dimethyldodecyl propionate	1811	2045
3-methyltridecyl propionate	1845	2078
11-methyltridecyl propionate	1876	2122

The mass spectrum of compound B (Fig. 3B) had diagnostic fragment ions *m/z* 168 (M^+^-74), 139, and 75, suggesting a propionate with a molecular weight of 242. The strong peak at *m/z* 139 in compound B suggested methyl branching on the 3rd and 9th carbon from the hydrocarbon end of the alcohol moiety. Therefore, we synthesized 2,8-dimethyldecyl propionate (2,8-DiMe-10Pr) and compared GC-MS data of compound B and 2,8-DiMe-10Pr. As expected, the mass spectrum of compound B and RI values on two columns (DB-5MS: 1610, DB-Waxetr: 1831) were identical to those of synthetic 2,8-DiMe-10Pr (Table 1). The mean ratio of 2,10-DiMe-12Pr and 2,8-DiMe-10Pr was 8:2, and the amount of the major component 2,10-DiMe-12Pr was estimated to be 5 pg/female using the external standard method.

**Figure 4:**
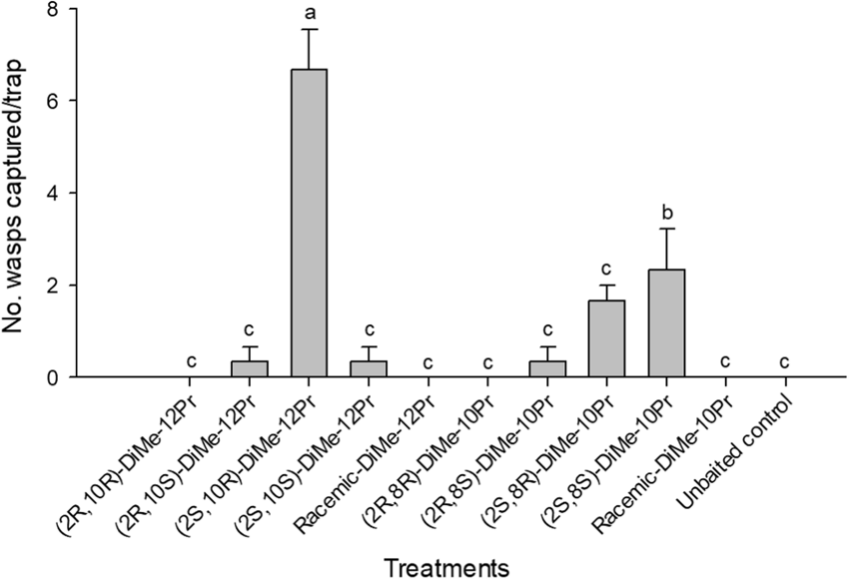
Number (mean + SE) of male *Eurytoma maslovskii* captured in traps baited with 100 μg of single enantiomers and racemic mixtures of 2,10-DiMe-12Pr and 2,8-DiMe-10Pr in Korean apricot orchards in Suncheon, Korea, on 3–5 May 2019 (*N* = 3 per treatment). There was statistical difference among treatments (Kruskal-Wallis analysis, *x*^2^ = 26.65, P = 0.003). Bars with the same letter are not significantly different based on pairwise comparisons using a Mann-Whitney U-test (*P* > 0.05).

**Figure 5:**
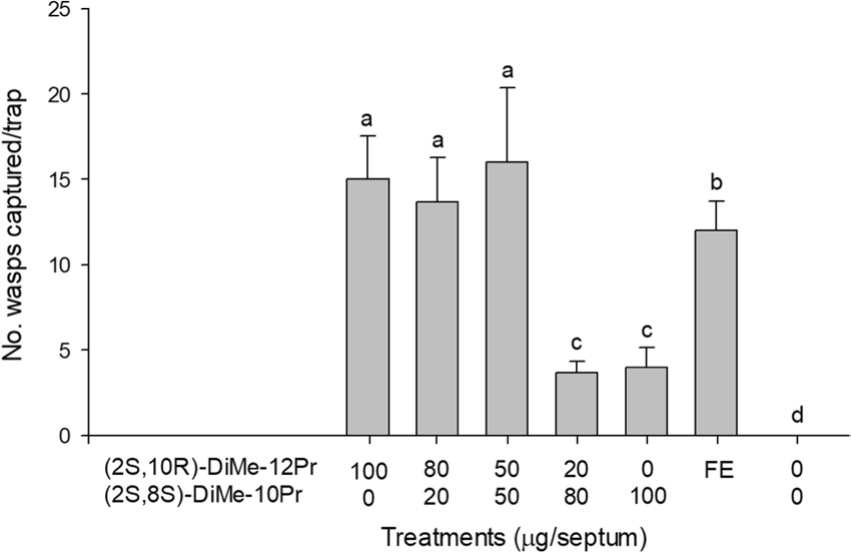
Number (mean + SE) of male *E. maslovskii* captured in traps baited with various ratios of (2*S*,10*R*)-DiMe-12Pr and (2*S*,8*S*)-DiMe-10Pr in Korean apricot orchards in Suncheon, Korea, on 9–11 May 2019 (*N* = 3 per treatment). There was statistical difference among treatments (Kruskal-Wallis analysis, *x*^2^ = 16.11, P = 0.0132). Bars with the same letter are not significantly different based on pairwise comparisons using a Mann-Whitney U-test (*P* > 0.05). FE = 20 female equivalents.

**Figure 6:**
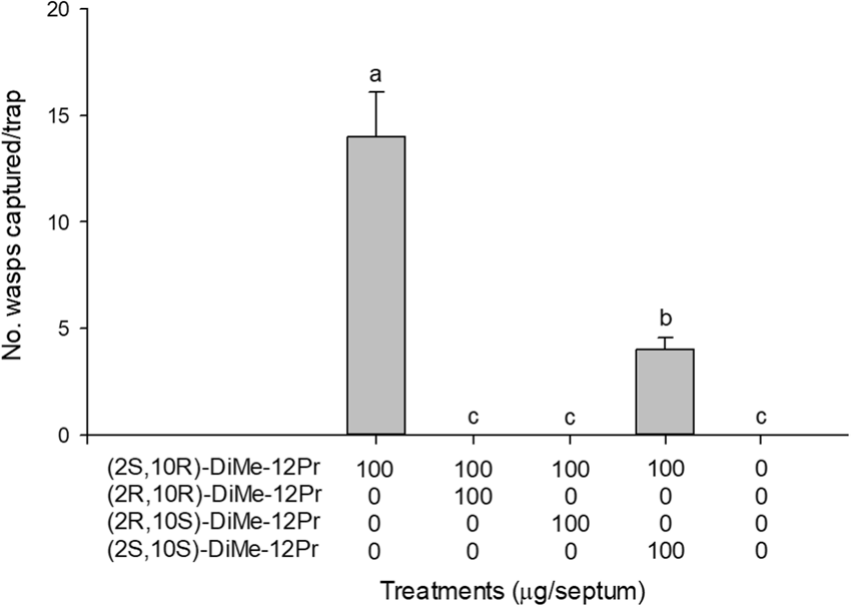
Number (mean + SE) of male *E. maslovskii* captured in traps baited with mixtures of (2*S*,10*R*)-DiMe-12Pr and other enantiomers in Korean apricot orchards in Suncheon, Korea, on 12–14 May 2019 (*N* = 3 per treatment). There was statistical difference among treatments (Kruskal-Wallis analysis, *x*^2^ = 13.75, P = 0.008). Bars with the same letter are not significantly different based on pairwise comparisons using a Mann-Whitney U-test (*P* > 0.05).

**Figure 7:**
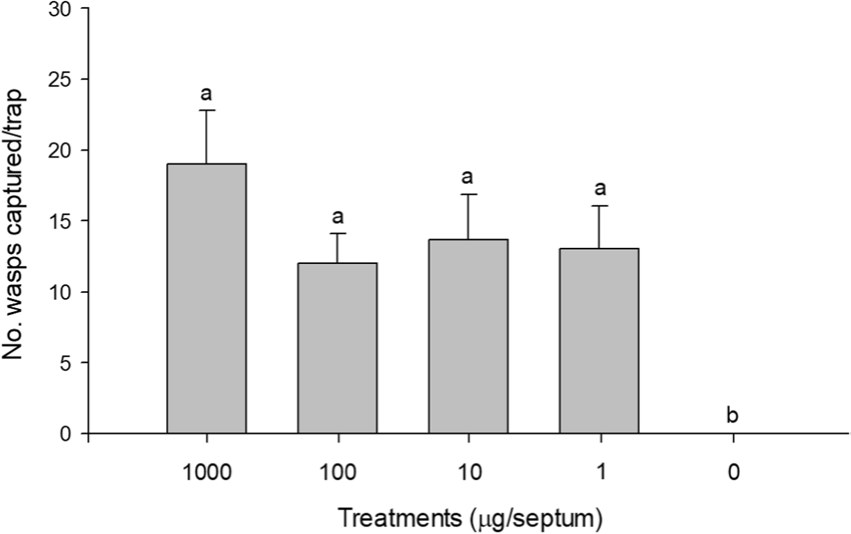
Number (mean + SE) of male *E. maslovskii* captured in traps baited with different doses of (2*S*,10*R*)-DiMe-12Pr in Korean apricot orchards in Suncheon, Korea, on 15–17 May 2019 (*N* = 3 per treatment). There was statistical difference among treatments (Kruskal-Wallis analysis, *x*^2^ = 9.54, P = 0.049). Bars with the same letter are not significantly different based on pairwise comparisons using a Mann-Whitney U-test (*P* > 0.05).

In experiment 2 compared the attractiveness of enantiomers of 2,10-DiMe-12Pr and 2,8-DiMe-10Pr, *E. maslovskii* males were most attracted to traps baited with (2 *S*,10 *R*)-DiMe-12Pr alone (Fig. 4). Traps baited with (2 *S*,8 *S*)-DiMe-10Pr caught significantly more males than unbaited control traps. Racemic samples that are mixtures of all four possible enantiomers were completely unattractive to males. Experiment 3, which tested the response of males to various blends (100:0, 80:20, 50:50, 20:80, 0:100) of (2 *S*,10 *R*)-DiMe-12Pr and (2 *S*,8 *S*)-DiMe-10Pr, revealed that the addition of (2 *S*,8 *S*)-DiMe-10Pr to (2 *S*,10 *R*)-DiMe-12Pr resulted in no significant increase in trap catches relative to (2*S*,10*R*)-DiMe-12Pr alone (Fig. 5). Traps baited with (2*S*,10*R*)-DiMe-12Pr alone attracted significantly more males than 20 female equivalents (FE). In experiment 4 testing the antagonist effects of different enantiomers of 2,10-DiMe-12Pr, adding a (2*R*,10*R*)- or (2*R*,10*S*)-isomer to the (2*S*,10*R*)-isomer completely inhibited the capture of males, and the addition of the (2*S*,10*S*)-isomer significantly reduced attraction (Fig. 6). Finally, in experiment 5 testing the sensitivity of the response to different amounts of the pheromone, equal numbers of males were attracted to all tested doses including a minimal dose of 1 μg of (2*S*, 10*R*)-DiMe-12Pr (Fig. 7).

## Discussion

In this study, we provide laboratory and field results demonstrating that (2*S*,10*R*)-DiMe-12Pr is the major component of the sex pheromone of *Eurytoma maslovskii*. This is the first case in which a long-range, female-derived sex pheromone of a species from Chalcidoidea is characterized under field conditions.

The long-range sex pheromones identified for Hymenoptera to date exhibit a wide range of molecular structures such as carboxylic acids, carboxylic esters, aldehydes, pyrazines, and pyran^2^. Among the carboxylic esters identified are 3,7-dimethylpentadecan-2-yl acetate in *Neodiprion lecontei* (Fitch) (Diprionidae)^4^, ethyl-(*Z*)-9-hexadecenoate in *Syndipnus rubiginosus* Walley (Ichneumonidae)^6^, *Z*9-octadecen-4-olide in *Janus integer* Norton (Cephidae)^9^, and esters of 3,7-dimethyltridecan-2-ol in *Diprion pini* (Diprionidae)^10^. Propionates of secondary alcohols with two or three methyl branches in C_13_ and C_15_ chains have been reported as sex pheromone^10^ or attractants of the diprionid sawflies^25–27^. In addition, the western corn root worm, *Diabrotica virgifera virgifera* LeConte (Coleoptera: Chrysomelidae), uses 8-methyldecan-2-yl propionate as a sex pheromone^28^. However, the pheromone of *E. maslovskii* is the first propionate of branched primary alcohol identified as an insect pheromone, adding a new facet to insect pheromone chemistry.

The dimethyl alcohol precursors of active acetates or propionates reported from sawflies have intervals between methyl branch points of 3 methylenes^4,26^. Moreover, the most common dimethylalkanes observed in insects have 3-methylene interruptions, with 7-, 9-, and 11- bridges being the less common^29^. Thus, the major component of the sex pheromone of *E. maslovskii* having intervals of seven methylenes is unusual among insect semiochemicals reported so far.

We showed that female *E. maslovskii* contain only approximately 5 pg of pheromone, which is much less than the nanogram^5,6^ and microgram^13^ quantities of sex pheromones reported for other hymenopteran species. This is in agreement with the field-trapping tests that indicated that the pheromone attracted conspecific males at very low dose, revealing the pheromone’s potency and the antennae’s sensitivity to it. Various propionates or acetates of secondary alcohols with one, two, or three methyl branch(es) in a C_11_–C_15_ chain are known to be sex attractants of sawflies in Hymenoptera^30^. However, functionally active compounds in female extracts have been identified in only one species. Namely, Jewett *et al.*^4^ found that *N. lecontei* females contain approximately 150 pg of 3,7-dimethylpentadecen-2-yl acetate. Males of *Diprion similis* were successfully caught with traps baited with crude virgin female pheromone extract in field conditions^31^. However, there have been no demonstrations of the presence of the active component(s) in *D. similis* females, although there is evidence from field tests^25^ of males being attracted to the (2*S*,3*R*,7*R*)-isomer of the propionate of 3,7-dimethylpentadecen-2-ol, which was found in female body. These results suggest that the small quantity present explains why isolation and identification of sex pheromones of sawflies and wasps using methyl-branched esters as active compounds had proven difficult.

Field bioassays with the enantiomers of 2,10-DiMe-12Pr showed that males were selectively attracted by the (2*S*,10*R*)-isomer. Moreover, the addition of the other enantiomers to the (2*S*,10*R*)-isomer significantly decreased attraction of conspecific males, showing that the pheromone receptor neurons are highly specific for chiral compounds in males of this species. In particular, the addition of enantiomers with *R* configuration at the 2 position to the major pheromone component completely inhibited the attractiveness of the pheromone, suggesting that the chirality of sex pheromones may potentially contribute to premating reproductive isolation of closely related sympatric species. This would be a very interesting question to be addressed in a future study. The enantioselective response to methyl-branched propionates and acetates has been demonstrated in several sawfly species, including *Neodiprion lecontei*^32^, *Neodiprion sertifer*^33^, *Diprion similis*^25^ and *Diprion pini*^26^. These studies suggest that both female signaling and male response traits may have evolved to contribute to species-specific sexual communication in Hymenoptera.

2,8-DiMe-10Pr was identified as a minor component of female *E. maslovskii* thoracic extracts, but the behavioral role of this component remains unclear. The (2*S*,8*S*)-isomer was active as single components, although they were significantly less attractive than the major component, (2*S*,10*R*)-DiMe-12Pr. However, we did not find any significant behavioral effect of the most attractive (2*S*,8*S*)-enantiomer when it was combined with (2*S*,10*R*)-DiMe-12Pr at the level (ca. 20%) found in the female extracts, indicating that (2*S*,8*S*)-DiMe-10Pr cross-activated the peripheral pheromone receptor for (2*S*,10*R*)-DiMe-12Pr^34^. Therefore, enantiomers of 2,8-DiMe-10Pr found in female *E. maslovskii* thoracic extracts may just be present as by-products of the pheromone biosynthetic pathway (e.g., *β*-oxidation^35^) that produces the major component. On the other hand, this minor component may serve an antagonistic function to suppress attraction of sympatric species, thereby maximizing prezygotic mating barriers (e.g., *Adoxophyes* spp.^36^). Future research should aim to determine the biosynthetic pathway of the methyl-branched esters and identify the sex pheromones of sympatric hymenopteran species. Such studies will help to clarify the function of this minor component.

## Methods

### Insect material

Mummified fruits containing diapausing larvae of *Eurytoma maslovskii* were collected from Korean apricot (*Prunus mume*) orchards in Suncheon, Korea (34.9 N, 127.4E) in January and February 2016, 2017, and 2018. The infested fruits were placed in screen cages (30 × 30 × 30 cm), and maintained at 25 °C under a photoperiod of L14:D10. Newly emerged adults were collected daily, and the sexes were separated based on the presence or absence of a long ovipositor on the ventral side of the abdomen. The adults were kept individually in plastic bottles (7 cm height, 2.5 cm diameter) and provided with a cotton pad soaked with a 10% sucrose solution.

### Chemicals

Racemic 3-methyltridecyl propionate (90% purity), 11-methyltridecyl propionate (91% purity), 2,10-dimethyldodecyl propionate (2,10-DiMe-12Pr) (99% purity), 4,10-dimethyldodecyl propionate (91% purity), 6,10-dimethyldodecyl propionate (92% purity), 3,7-dimethyldodecyl propionate (90% purity), 3,9-dimethyldodecyl propionate (91% purity), and 2,8-dimethyldecyl propionate (2,8-DiMe-10Pr) (99% purity) were synthesized by one of the authors (KBK). The corresponding alcohols were synthesized by bromination of the commercially available methylated alkanol or methylated alkandiol, followed by chain extension with the appropriated tetrahydropyran (THP)-protected alkylmangesium bromide^37,38^. The propyl esters were prepared with the corresponding alcohol and propionyl chloride. All structures were confirmed by NMR analysis and/or GC-MS.

#### 2,10-dimethyldodecyl propionate

^1^H NMR (500 MHz, CDCl_3_) δ 3.958 (dd, *J* = 10.7, 5.9 Hz, 1H), 3.853 (dd, *J* = 10.7, 6.9 Hz, 1H), 2.332 (q, *J* = 7.6 Hz, 2H), 1.819–1.718 (m, 1H), 1.420–1.202 (m, 14H), 1.147 (t, *J* = 7.6 Hz, 3H), 1.157–1.049 (m, 3H), 0.917 (d, *J* = 6.8 Hz, 3H), 0.895 (t, *J* = 7.3 Hz, 3H), 0.839 (d, *J* = 6.4 Hz, 3H). ^13^C NMR (126 MHz, CDCl_3_) δ 174.65 (C), 69.33 (CH_2_), 36.65 (CH_2_), 34.42 (CH), 33.39 (CH_2_), 32.57 (CH), 30.00 (CH_2_), 29.86 (CH_2_), 29.66 (CH_2_), 29.51 (CH_2_), 27.67 (CH_2_), 27.11 (CH_2_), 26.84 (CH_2_), 19.23 (CH_3_), 16.90 (CH_3_), 11.42 (CH_3_), 9.22 (CH_3_).

#### 2,8-dimethyldecyl propionate

^1^H NMR (500 MHz, CDCl_3_) δ 3.958 (dd, *J* = 10.7, 5.9 Hz, 1H), 3.854 (dd, *J* = 10.7, 6.9 Hz, 1H), 2.332 (q, *J* = 7.6 Hz, 2H), 1.820–1.705 (m, 1H), 1.412–1.214 (m, 10H), 1.146 (t, *J* = 7.6 Hz, 3H), 1.157–1.048 (m, 3H), 0.918 (d, *J* = 6.7 Hz, 3H), 0.854 (t, *J* = 7.3 Hz, 3H), 0.840 (d, *J* = 6.3 Hz, 3H). ^13^C NMR (126 MHz, CDCl_3_) δ 174.64 (C), 69.32 (CH_2_), 36.60 (CH_2_), 34.41 (CH), 33.40 (CH_2_), 32.58 (CH), 30.19 (CH_2_), 29.51 (CH_2_), 27.67 (CH_2_), 27.04 (CH_2_), 26.87 (CH_2_), 19.23 (CH_3_), 16.91 (CH_3_), 11.41 (CH_3_), 9.22 (CH_3_).

Moreover, all four possible enantiomers (>99% ee) of 2,10-DiMe-12Pr and 2,8-DiMe-10Pr used in field bioassays were synthesized by one of the authors (KM). Details of the syntheses of these chiral compounds will be described elsewhere (Okubo *et al*., in press).

### Pheromone extraction

Preliminary observations revealed that *E. maslovskii* usually mates in the field during the morning, usually at 0900–1200 h. Males were captured in traps baited with female thoraces without heads or abdomens, as in other eurytomid wasp^20^. For this, 1–2-d-old females were sectioned into head, thorax and abdomen under a binocular microscope during the fourth to fifth hour of the photophase. Ten excised thoraces were immersed in 200 μl hexane in a 5-ml conical glass vial (Wheaton, Millville, NJ, USA) for 10 min and pooled to yield a crude extract. The hexane extract was then transferred into another vial and stored at −20 °C until use.

### Fractionation of thoracic extracts

A column prepared from a Pasteur pipette plugged with a small plug of glass wool was loaded with 500 mg silica gel (Wakogel C-200, Japan), rinsed with hexane, and loaded with a thoracic extract from 500 virgin females. Substances were then eluted from the column sequentially with 4 ml hexane (hexane fraction), 4 ml 1% diethyl ether in hexane (1% fraction), 4 ml 5% diethyl ether in hexane (5% fraction), 4 ml 30% diethyl ether in hexane (30% fraction), and 4 ml diethyl ether (ether fraction). Sequential elution with hexane, diethyl ether in hexane, and diethyl ether yielded fractions containing nonpolar, modest polar and more polar compounds, in sequence. Each fraction was concentrated to 50 μl under a stream of nitrogen and kept in a freezer (−20 °C) until being used in field bioassays or chemical analyses.

### Gas Chromatography-Electroantennographic Detection (GC-EAD)

Aliquots of behaviorally active fractions (5% ether in hexane) of female thoracic extracts were subjected to gas chromatography-electroantennographic detection (GC-EAD) analyses^39^, using an Agilent GC 7890B, equipped with a DB-5 column (30 m×0.25 mm ID, 0.25 μm film thickness; J&W Scientific, CA, USA) and an electroantennography system (Syntech, Kirchzarten, Germany). The injector and detector temperatures were 250 and 260 °C, respectively. The effluent from the GC column was split between a flame ionization detector (FID) and the antenna of a male wasp (split ratio 1:1). Hydrogen was used as the carrier gas. The GC oven was programmed from 80 °C/1 min to 250 °C at 10 °C/min and held for 10 min.

An excised male *E. maslovskii* antenna was positioned between two glass capillary electrodes. Each end of the antenna was embedded in electrode gel (spectra 360; Parker Laboratories, NJ, USA) and applied on electrode holders. The EAD exit port temperature was maintained at 230 °C and the antennal preparation was continuously exposed to a charcoal-filtered and humidified airstream. Antennal signals were amplified using a universal AC/DC amplifier and analyzed on a computer equipped with a signal acquisition interface board (IDAC-4, Syntech) running a GC-EAD software (AutoSpike version 3.2, Syntech).

### Gas Chromatography-Mass Spectrometry (GC-MS)

Female extracts and synthetic standards were analyzed on an Agilent 6890 N GC interfaced to an Agilent 5975 C mass-selective detector as previously described^40^. Samples were run on a non-polar DB-5MS or polar DB-Waxetr column (30 m×0.25 mm ID, 0.25 μm film thickness; J&W Scientific, CA, USA). For the DB-5MS column, the oven temperature was identical to that previously described for the GC-EAD system. For the DB-Waxetr column, the oven temperature was maintained at 80 °C for 1 min, increased to 180 °C at 10 °C/min, then to 220 °C at 5 °C/min, and held for 10 min. The injection was splitless, and helium was the carrier gas (1 ml/min). The injector and transfer line temperatures were 250 °C. Electron ionization mass spectra were recorded from *m/z* 30 to 350 at 70 eV, with the ion source temperature of 230 °C. GC retention times are quoted as retention indices (RIs) relative to those of *n*-alkanes. Compounds from female thoracic extracts were identified by comparisons of their RIs and mass spectra with those of authentic standards on the DB-5MS and DB-Waxetr columns. Quantities of each compound in the female extracts were calculated using hexane solution (1 ng/uL) of synthetic racemic 2,10-DiMe-12Pr as an external standard.

### Field trials

Field experiments were conducted in three Korean apricot orchards in Suncheon, Korea in April and May 2018 and 2019. Delta traps (Green Agro Tech, Gyeongsan, Korea) were baited with red rubber septa (Aldrich Chemical, Milwaukee, WI, USA) loaded with test compounds. Traps were hung from apricot branches approximately 1.5 m above ground. In all experiments, a randomized complete block design was used with three replicates per treatment. The distance between traps within each block was 10 m, and the distance between blocks was at least 100 m.

Experiment 1 tested biological activities of five fractions of female thoracic extracts. Traps were baited with 50 female equivalents of the fractions. In addition, traps baited with crude extracts of 50 female thoraces and traps without baits were used as controls. Experiment 2 was designed to determine which enantiomers of 2,10-DiMe-12Pr and 2,8-DiMe-10Pr are pheromone components of *E. maslovskii*. Treatments were racemic mixtures, (*RR*)-, (*RS*)-, (*SR*)-, and (*SS*)-enantiomers of 2,10-DiMe-12Pr and 2,8-DiMe-10Pr, respectively. Experiment 3 analyzed the potential synergism between (2*S*,10*R*)-DiMe-12Pr and (2*S*,8*S*)-DiMe-10Pr by testing them separately and in mixtures with different mixing ratios (100:0, 80:20, 50:50, 20:80, 0:100). Experiment 4 was designed to determine the antagonist effects of the other enantiomers (*RR*, *RS*, or *SS*) on the attraction to (2*S*,10*R*)-DiMe-12Pr. Finally, experiment 5 compared the relative attractiveness of different doses (1000, 100, 10, 1 ug) of (2*S*,10*R*)-DiMe-12Pr.

Because field trapping data did not adequately meet assumptions of normality or heteroscedasity, differences were analyzed via nonparametric Kruskal-Wallis analysis of variance (SAS Institute Inc. 2014). Post-hoc pairwise comparisons were computed using a Mann-Whitney U-test at the 5% significance level.
